# The Nlrp3 inflammasome as a “rising star” in studies of normal and malignant hematopoiesis

**DOI:** 10.1038/s41375-020-0827-8

**Published:** 2020-04-20

**Authors:** Mariusz Z. Ratajczak, Kamila Bujko, Monika Cymer, Arjun Thapa, Mateusz Adamiak, Janina Ratajczak, Ahmed K. Abdel-Latif, Magda Kucia

**Affiliations:** 1 Stem Cell Institute at James Graham Brown Cancer Center, University of Louisville, Louisville, KY USA; 2 Department of Regenerative Medicine, Center for Preclinical Research and Technology, Medical University of Warsaw, Warsaw, Poland; 3 Division of Cardiovascular Medicine, Gill Heart Institute, University of Kentucky, Lexington, KY USA

**Keywords:** Stem cells, Cell biology

## Abstract

Recent investigations indicate that hematopoiesis is coregulated by innate immunity signals and by pathways characteristic of the activation of innate immunity cells that also operate in normal hematopoietic stem progenitor cells (HSPCs). This should not be surprising because of the common developmental origin of these cells from a hemato/lymphopoietic stem cell. An important integrating factor is the Nlrp3 inflammasome, which has emerged as a major sensor of changes in body microenvironments, cell activation, and cell metabolic activity. It is currently the best-studied member of the inflammasome family expressed in hematopoietic and lymphopoietic cells, including also HSPCs. It is proposed as playing a role in (i) the development and expansion of HSPCs, (ii) their release from bone marrow (BM) into peripheral blood (PB) in stress situations and during pharmacological mobilization, (iii) their homing to BM after transplantation, and (iv) their aging and the regulation of hematopoietic cell metabolism. The Nlrp3 inflammasome is also involved in certain hematological pathologies, including (i) myelodysplastic syndrome, (ii) myeloproliferative neoplasms, (iii) leukemia, and (iv) graft-versus-host disease (GvHD) after transplantation. The aim of this review is to shed more light on this intriguing intracellular protein complex that has become a “rising star” in studies focused on both normal steady-state and pathological hematopoiesis.

## Introduction

Several members of the family of intracellular inflammasome protein complexes have been identified that have pro- or even anti-inflammatory functions [1–6]. The Nlrp3 inflammasome is, so far, the best-studied of these multiprotein complexes, consisting of Nlrp3 protein, apoptosis-associated speck-like protein containing a CARD (ASC), and procaspase-1 [4, 7–9]. This intriguing protein complex is located in the cytoplasm in an inactive form. Upon activation, it becomes an aggregate composed of several Nlrp3 molecules (speck complexes), each containing Nlrp3 protein, ASC, and procaspase-1. Importantly, upon inflammasome activation, procaspase-1 protein becomes cleaved to functional caspase-1, whose main function is conversion of the inactive and intracellularly potent proinflammatory cytokines pro-IL-1β and pro-IL-18 into their active forms [10, 11]. Mature IL-1β and IL-18 are than secreted from the cells [12]. The pleiotropic effects of these cytokines in hematopoiesis, aging, and metabolic complications were studied in the past but have currently become even more interesting after the discovery that their activation occurs in an Nlrp3 inflammasome-dependent manner [1–3].

The expression of the Nlrp3 inflammasome has been primarily described in innate immunity cells, including monocytes, macrophages, granulocytes, and dendritic cells [13–18]. Subsequently, this protein complex was also found to be present in T and B lymphocytes [19–22]. However, more significant are our recent results demonstrating the expression of the Nlrp3 inflammasome in postnatal murine and human hematopoietic stem progenitor cells (HSPCs) [23]. This fact should not be surprising, taking into consideration the common origin of all these cells from the stem cell that is at the top of the hierarchy for hemato/lymphopoietic lineages [24].

**Fig. 1:**
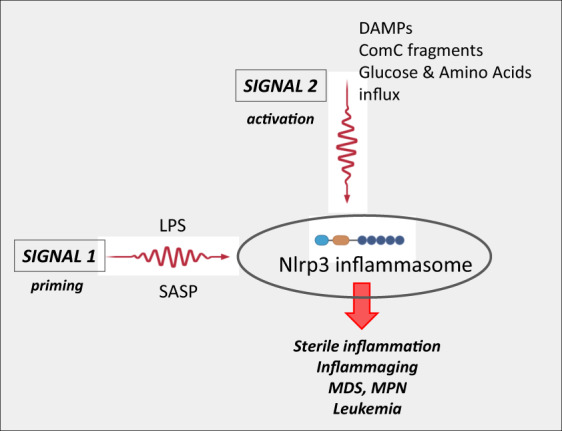
Priming and activation of Nlrp3 inflammasome by “Signal 1” and “Signal 2,” respectively. The important priming factors that deliver “Signal 1” are (i) intestinal bacteria-derived liposaccharide (LPS) and (ii) senescence-associated secretory phenotype (SASP) cytokines such as TNF-α and IL-6. “Signal 2” that activates Nlrp3 inflammasome is delivered by danger-associated molecular pattern molecules (DAMPs) or alarmines including eATP, HMGB1, S100A8/A9, uric acid crystals, extracellular DNA and mRNA complexes, ComC cleavage fragments (C3a, C5a, C5b–C9) and glucose/amino acid influx. In response to these signals Nlrp3 inflammasome mediates sterile inflammation in hematopoietic tissues and inflammaging that may promote myelodysplasia (MDS), myeloproliferative neoplasms (MPN), and leukemia.

The basic cellular expression of Nlrp3 inflammasome elements is regulated under steady-state conditions by a priming signal described in the literature as “signal 1” (Fig. 1) [4, 25, 26]. This signal is continuously delivered to the cells by liposaccharide (LPS) released from intestinal Gram-negative bacteria after engaging Toll receptor 4 (TLR4) [27]. This interaction leads to the transcription of Nlrp3 inflammasome components in an NF-κb transcription factor-dependent manner and its basic level in the cells. This interplay between intestine-derived LPS and baseline expression of the Nlrp3 inflammasome became somewhat more relevant in light of recent intensive studies on the role of the intestinal microbiome (microbiota) in body homeostasis [28, 29]. “Signal 1” could also be delivered to the innate immunity cells after stimulation by TNF-α or IL-6 (Fig. 1). Both of these cytokines are principal members of the family of proinflammatory mediators, referred to collectively in the literature as the senescence-associated secretory phenotype (SASP), and are implicated in priming the expression of the Nlrp3 inflammasome with advancing age [30].

In contrast to priming “signal 1,” functional activation of the synthesized Nlrp3 inflammasome is mediated by “signal 2,” which is delivered by exogenous or endogenous danger-associated products related to infection, cell activation, or cell/tissue damage [31–34] (Fig. 1). These activating signals are classified into (i) exogenous pathogen-associated molecular pattern molecules (PAMPs), which are microbial, fungal, viral, and parasitic products released during infection and, what is more important for the topic of this review, also by (ii) endogenous danger-associated molecular pattern molecules (DAMPs), also known as alarmines that are released during nonpathogen-related “sterile inflammation” [35]. DAMPs, which are secreted after cell activation or damage, include (i) extracellular adenosine triphosphate (eATP), which is a principal mediator of purinergic signaling, (ii) reactive oxygen species (ROS), (iii) the nuclear protein high mobility group protein B1 (HMGB1), (iv) a multigenic family of calcium-modulated proteins, including S1009a and S1008a, (v) uric acid crystals, and (vi) extracellular DNA and RNA fragments [4, 36–40].

It is widely accepted that, as activators of the Nlrp3 inflammasome, DAMPs are involved in producing as mentioned above “sterile inflammation,” which is seen in tissues in stress situations and in tissue/organ damage in the absence of infection-related PAMPs [35, 41–43]. Sterile inflammation in bone marrow (BM) is also induced during pharmacological mobilization of HSPCs after systemic administration of granulocyte colony-stimulating factor (G-CSF) or the CXCR4 receptor antagonist (AMD3100) [23, 44]. It also occurs after myeloablative conditioning for hematopoietic transplantation after administration of high doses of cytostatics or total body irradiation [45, 46]. Pharmacological mobilizations as well as myeloablative conditioning for hematopoietic transplantation induce the Nlrp3 inflammasome, both in hematopoietic cells and in the BM microenvironment.

At the molecular level, the Nlrp3 inflammasome is triggered in cells in response to a DAMPs-mediated calcium influx or potassium efflux but in addition—what is very exciting and was recently proposed in the case of human T lymphocytes—in response to changes in glucose and amino acid uptake [47]. This finding establishes an additional role for the Nlrp3 inflammasome as a sensor of metabolic activity in immune cells and possibly also in HSPCs. This effect explains, at least partially, the involvement of this protein complex in the proliferation and expansion of HSPCs, as these processes require energy release.

In this review we will briefly summarize the multiple effects of the Nlrp3 inflammasome in (i) development and expansion of HSPCs, (ii) mobilization of HSPCs, (iii) homing and engraftment of HSPCs after transplantation, (iv) HSPC aging (inflammaging) and the metabolism of immune cells (metaflammation), (v) myelodysplastic syndrome (MDS), (vi) myeloproliferative neoplasms (MPN) and leukemia, and finally (vi) posttransplantation graft-versus-host disease (GvHD).

An important step forward in modulating expression of the Nlrp3 inflammasome in cases in which its overexpression is unwanted has been the development of the small-molecule inhibitor MCC950 [48], which after successful testing in animals models awaits its first clinical trials in humans.

## Does Nlrp3 inflammasome signaling contribute to the development and expansion of HSPCs?

Based on the foregoing, this is a legitimate question to ask. In fact, an indication that Nlrp3 inflammasome signaling may indeed play a role in the developmental expansion of HSPCs is found in a recent report showing that glucose influx to the developing vertebrate embryo expands HSPCs in hematopoietic organs, and this effect depends on Nlrp3 inflammasome activation and IL-1β release [49]. As the authors discovered, glucose influx into HSPCs increases the release of IL-1β, and this effect was suppressed in IL-1β-KO cells and negatively affected the glucose flux effect on the number of murine early CD41^+^ HSPCs [49]. In support of a role for the Nlrp3 inflammasome, the authors observed that loss of its components prevented proliferation of embryonic HSPCs [49]. Moreover, when human iPSC-derived hemogenic cells were exposed to Nlrp3 inflammasome activators, there was a significant increase in multilineage hematopoietic colony formation. These results strongly suggest that the Nlrp3 inflammasome regulates expansion of early-development HSPCs [49].

Based on these provocative findings, further studies are needed to assess whether, in addition to embryonic HSPCs and iPSC-derived hemogenic cells [49], this early developmental role of the Nlrp3 inflammasome is preserved in postnatal normal murine and human HSPCs. If this scenario is confirmed, stimulation of Nlrp3 inflammasome activity may become an adjuvant strategy to improve ex vivo expansion of HSPCs. Our unpublished results indicate that, in fact, Nlrp3-KO mice have ~20% fewer Sca-1^+^Kit^+^Lin^–^ HSPCs in BM than do WT animals.

Another interesting candidate for augmenting Nlrp3 inflammasome–IL-1β effects could be IL-1α (also known as hemopoietin 1), which is structurally related to IL-1β [50]. However, these cytokines differ in their expression. While IL-1β expression is induced in innate immunity cells in a transcription factor NF-κB-dependent manner after exposure to DAMPs, IL-1α is also synthesized continuously as a precursor protein and stored in the cytoplasm of BM cells of mesenchymal origin [12]. It has been reported that calpain, which is a calcium-activated cysteine protease that is associated with the plasma membrane, is primarily responsible for the cleavage of the IL-1α precursor into a mature molecule [50]. It has also been proposed that there is an additional role played by caspase-1 [51].

Surprisingly, more detailed experimental results for hematopoiesis have so far been generated with IL-1α. Nevertheless, since both IL-1β and IL-1α bind to a common receptor (IL-1R) that is present on a variety of target cells, their biological effects are similar [52]. Specifically, the activation of IL-1R induces the production by accessory cells of hematopoietic cytokines that stimulate granulocyto/monopoiesis and synergize with other colony-stimulating factors in the proliferation of HSPCs [53]. In vivo IL-1α administration accelerates hematopoietic reconstitution in mice after chemotherapy in a model of radiation-induced myelosuppression [54]. Based on this finding, one should expect similar effects after administration of Nlrp3 inflammasome-derived IL-β.

Moreover, since the Nlrp3 inflammasome is strongly activated by the extracellular purine nucleotide eATP, these effects on the HSPC response depend on purinergic signaling in which eATP is the principal stimulatory mediator [44, 55–57]. The role of purinergic signaling in hematopoietic development has been convincingly demonstrated by another group in a zebra fish model in the context of the prohematopoietic effects of the eATP metabolite extracellular adenosine (eAdo) [58].

This interesting effect of the Nlrp3 inflammasome on hematopoiesis needs to be better addressed in models of stress-induced hematopoiesis. For example, do Nlrp3 inflammasome-KO animals display a defect in hematopoietic recovery from sublethal irradiation? Moreover, if any defects are observed, would they depend on Nlrp3 inflammasome expression in hematopoietic cells or in the hematopoietic microenvironment? Which signaling pathways and transcription factors are involved in these phenomena? To address these questions, we are currently performing appropriate experiments.

**Fig. 2:**
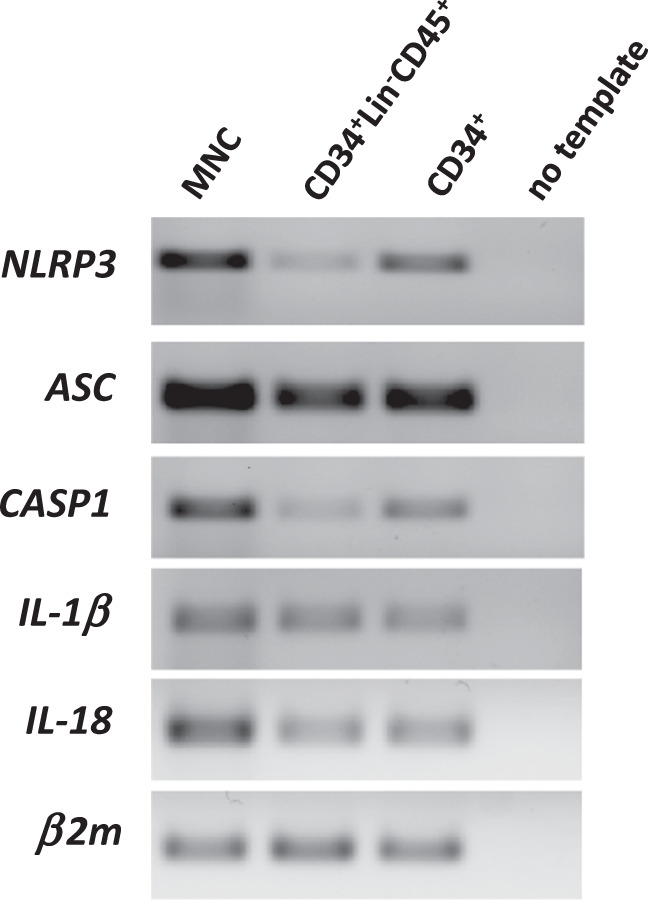
Expression of Nlrp3 complex mRNAs in HSPCs. The expression of inflammasome genes was detected in purified mRNA in human CD34^+^ and CD34^+^lin^−^CD45^+^ cells by reverse transcription polymerase chain reaction (RT-PCR). Samples containing only water instead of cDNA and samples without reverse transcriptase were used in each run as negative controls. Representative agarose gels of the RT-PCR amplicons are shown.

Figure 2 shows the expression of inflammasome components in purified human cells. The detectable mRNA expression of Nlrp3 inflammasome components in CD34^+^lin^–^CD45^+^ cells indicates that the biology of adult HSPCs is also directly affected by endogenous Nlrp3 inflammasome activation. As reported recently, the sensing of glucose and/or amino acid influx into cells by the Nlrp3 inflammasome results in adult human lymphocytic cell proliferation and differentiation [49]. Does a similar effect occur in human HSPCs as well?

Finally, it would be interesting to address whether, in addition to IL-1β, another Nlrp3 inflammasome-activated cytokine, such as IL-18, is released and processed to its active form by caspase-1 and plays a role in hematopoiesis. However, as of today, IL-18 is considered mainly to be a proinflammatory cytokine that modulates both innate and adaptive immunity, being involved in the pathogenesis of autoimmune and inflammatory diseases [59–61].

## A novel role for the Nlrp3 inflammasome in the trafficking of HSPCs

Since egress of HSPCs into peripheral blood (PB) is mediated by induction of a state of sterile inflammation in the BM hematopoietic microenvironment due to innate immunity activation [44], we became interested in whether the Nlrp3 inflammasome plays a significant role in the mobilization process [62]. We also asked whether Nlrp3 inflammasome-mediated mechanisms play a role in the mobilization of other BM-residing stem/progenitor cells, such as mesenchymal stromal cells, endothelial progenitor cells, and very small embryonic-like stem cells. In parallel, since sterile inflammation is also induced in the BM microenvironment after conditioning for hematopoietic transplantation by myeloablative radiochemotherapy, and the Nlrp3 inflammasome is expressed in HSPCs present in the hematopoietic graft (Fig. 2), we became interested in whether it plays a role in egress of these cells from BM into PB during mobilization process as well as in navigation of HSPCs toward chemoattractants expressed in the BM microenvironment.

### The role of the Nlrp3 inflammasome in stem cell mobilization

Our already published results demonstrated that the Nlrp3 inflammasome plays a crucial role in the mobilization into PB of HSPCs and other BM-residing stem cells [62]. In support of this finding, the Nlrp3 inflammasome becomes activated during mobilization, both in innate immunity cells (granulocytes, monocytes, and dendritic cells) and in HSPCs in response to G-CSF or AMD3100. This activation occurs in a paracrine/autocrine manner in response to DAMPs, including extracellular alarmines such as eATP, HMGB1, and S100 proteins [23]. Effector cells of innate immunity that are activated by these factors secrete higher levels of alarmines and amplify sterile inflammation in the BM microenvironment by employing positive feedback loops. It is worthwhile addressing again the important role of “signal 1” in the baseline expression of the Nlrp3 inflammasome provided by LPS derived from intestinal Gram-negative bacteria [10, 63]. To confirm the role of LPS in priming the mobilization process, it has been shown that mice depleted by antibiotics of LPS-producing intestinal bacteria are rendered poor mobilizers [64].

We have recently proposed that eATP-induced mobilization of HSPCs couples Nlrp3 inflammasome purinergic signaling with activation of the complement cascade (ComC) and release of ComC cleavage fragments, including C3- and C5-derived C3a and C5a anaphylatoxins, respectively [23, 44, 65, 66]. The Nlrp3 inflammasome is then activated in a positive feedback manner by C3a and C5a anaphylatoxins, which maintains a sterile inflammation state in the BM microenvironment. In addition, the Nlrp3 inflammasome may also become activated by the C5b–C9 sublytic membrane attack complex. On the other hand, DAMPs secreted from innate immunity cells after Nlrp3 inflammasome activation may in turn activate the ComC. Evidence has accumulated that ComC cleavage fragments are crucial for egress of HSPCs from BM into PB [67–69].

**Fig. 3:**
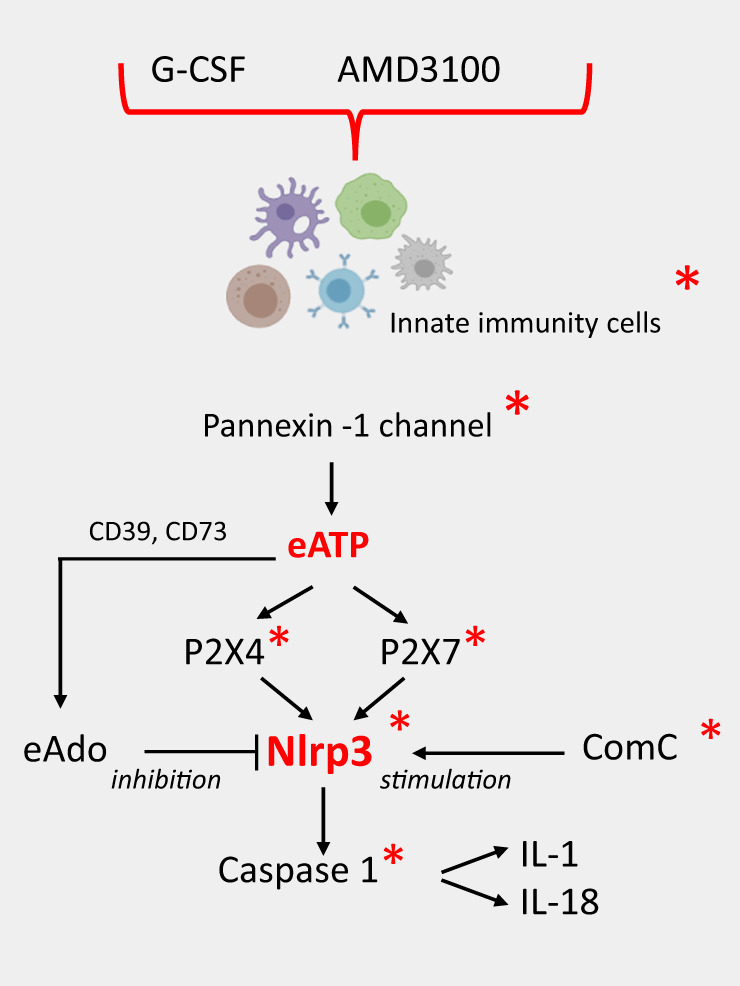
The purinergic signaling–Nlrp3 inflammasome–ComC network in the mobilization of HSPCs. Promobilizing agents stimulate the release of eATP from innate immunity cells (granulocytes, monocytes, and dendritic cells) in a pannexin-1-channel-dependent manner. eATP activates the Nlrp3 inflammasome in these cells via the P2X4 and P2X7 receptors in an autocrine/paracrine manner, which subsequently activates caspase-1 to release active IL-1β and IL-18. Autocrine/paracrine stimulation of innate immunity cells by both of these cytokines leads to release of several other DAMPs and activation of the ComC. As we propose, IL-1β and IL-18 form an endogenous positive feedback promobilization “machinery” in HSPCs. Red asterisks indicate elements whose attenuation results in poor mobilization in our recently published or preliminary results. This process is negatively regulated by the anti-inflammatory action of eATP metabolite extracellular adenosine (eAdo) that activates heme oxygenase 1 (HO-1) that inhibits Nlrp3 inflammasome. The metabolism of eATP to Ado is mediated by the cell-surface ectonucleotidases CD39 and CD73.

Figure 3 depicts the crucial elements of the purinergic signaling–Nlrp3 inflammasome–ComC axis identified so far that play a role in the HSPC mobilization process. Deficiency of the elements highlighted by asterisks leads to defective activation of the Nlrp3 inflammasome or to defective execution of its downstream effects and correlates with poor HSPCs mobilization status. What is not shown in this scheme, optimal mobilization requires first “signal 1” to prime the Nlrp3 inflammasome via TLR4 receptors in innate immunity cells, where intestine Gram-negative bacteria-derived LPS plays an important role. The activation of “signal 2” is delivered by eATP released via pannexin-1 channels to activate P2X7 and P2X4 purinergic receptors on the hematopoietic cells. The proper expression of the Nlrp3 inflammasome complex and the activity of caspase-1 in releasing active IL-1β and IL-18 together with activated ComC products propagates the innate immunity-driven sterile inflammation state in the BM microenvironment. This process is negatively regulated by the eAdo [70] and, not shown in this scheme, by eAdo induced anti-inflammatory enzyme heme oxygenase 1 [71].

### Does Nlrp3 inflammasome activation engage involvement of endogenous promobilization factors?

To address this intriguing question, the role of IL-1β and IL-18 released from cells in a Nlrp3 inflammasome–caspase-1-dependent manner during mobilization requires special attention. These important proinflammatory cytokines, when injected into mice strongly induce sterile inflammation in BM and mobilize HSPCs [23]. Based on the abovementioned fact that IL-1β and IL-1α share the same receptor, IL-1R, it is not surprising that we recently observed similar promobilizing effects in mice after systemic administration of IL-1α (unpublished). More importantly, we recently observed an inhibition in the release of these cytokines from cells in caspase-1-KO animals, resulting in a significant decrease in egress of HSPCs into PB (Fig. 3). This result may indicate the presence of an endogenous Nlrp3 inflammasome–caspase-1–IL-1α/β and –IL-18 “promobilization mechanism” that is activated after pharmacological administration by G-CSF or the CXCR4 antagonist AMD3100, both of which strongly induce the Nlrp3 inflammasome and caspase-1 activities in innate immunity cells and HSPCs (manuscript submitted). In this context G-CSF and AMD3100, via induction of purinergic signaling and the ComC, induce sterile inflammation in BM and provide “signal 2” for Nlrp3 inflammasome activation. As a consequence, the release of IL-1β, IL-18, and IL-1α involves subsequently involvement of autocrine/paracrine feedback loops that potentiate this process [23].

### The role of the Nlrp3 inflammasome in homing and engraftment of HSPCs

As mentioned above, myeloablative conditioning for hematopoietic transplantation may also induce a state of sterile inflammation in the BM of the recipient. In addition, recent results from our laboratory indicate that the Nlrp3 inflammasome becomes activated in HSPCs harvested for transplantation. This activation plays an important, and so far, underappreciated, role in the homing of HSPCs to BM niches. Further supporting such a role, we recently found that HSPCs from Nlrp3-KO mice have defective migration in response to BM chemoattractants, including stromal-derived factor 1 (SDF-1) and eATP, which are both upregulated in the BM microenvironment after myeloablative conditioning for transplantation [72].

**Fig. 4:**
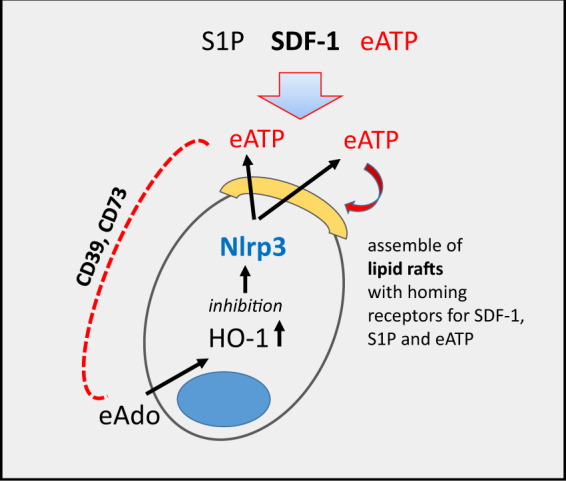
The role of eATP in the homing and engraftment of HSPCs. eATP plays a dual role in the homing of HSPCs to BM. On the one hand, eATP, whether autocrine-secreted from transplanted HSPCs or secreted in response to conditioning for transplantation from cells in the BM microenvironment, promotes formation of membrane lipid rafts (yellow cap), which assemble major chemoattractant receptors for HSPCs (SDF-1, S1P and eATP). Mobilization and homing of HSPCs are negatively controlled by the eATP metabolite eAdo due to upregulation of intracellular HO-1, which is an Nlrp3 inflammasome inhibitor. The metabolism of eATP to eAdo is mediated by the cell-surface ectonucleotidases CD39 and CD73.

To explain this interesting phenomenon, we propose the involvement of an autocrine or paracrine release of eATP from donor HSPCs, which enhances, in a membrane lipid raft formation-dependent manner, their responsiveness to BM chemotactic gradients (Fig. 4 and manuscript submitted). Indeed, incorporation of CXCR4 into membrane lipid rafts on the surface of HSPCs enhances their migration in response to a physiological SDF-1 gradient and enhances their seeding efficiency into BM after transplantation [73]. Lipid rafts provide better contact and interaction of the CXCR4 receptor with downstream signaling pathways involved in cell migration [73, 74]. In fact, we found that HSPCs from Nlrp3-KO mice have defective homing and engraftment in syngeneic wild type animals, and this defect is due to their defective lipid raft formation and migration in response to homing gradients (manuscript submitted).

However, further studies are needed to elucidate the role of the Nlrp3 inflammasome, as expressed in the donor BM microenvironment conditioned by myeloablative treatment for transplantation, in the homing and engraftment of transplanted cells. This topic will be studied in homing and engraftment experiments after transplantation of normal murine HSPCs into lethally irradiated Nlrp3-KO mice and control littermates.

## Involvement of the Nlrp3 inflammasome in the inflammaging of hemato/lymphopoiesis

Aging is an inevitable consequence of life and is most likely preprogrammed in the genes of all living organisms. It accelerates after reproductive age, when the genes have already been passed on to the next generation, and several mechanisms are currently proposed that accelerate this process [75, 76]. During aging, a state of chronic, low-grade systemic sterile inflammation develops [77]. One of the markers of inflammation is an increase in the plasma levels of the TNF-α and IL-6 proinflammatory mediators, which are components of the SASP and, as mentioned above, provide, besides LPS, “signal 1” for NF-κB-dependent transcription of Nlrp3 inflammasome elements in innate immunity cells and macrophages play here a central role [30].

The process of aging affects all tissues and organs, and the BM and hematopoiesis are not exceptions. Aging is characterized in the BM by enhanced myelopoiesis due to increased numbers of myeloid-biased HSPCs and myeloid cells, which leads to the dominance of hematopoiesis over lymphopoiesis. As a result of this shift, the pool of B and T lymphocytes shrinks over time in hematopoietic organs [78]. The increase in myelopoiesis can be explained, at least partially, by an increase in the release of Nlrp3 inflammasome-derived IL-1β and IL-1R signaling, which, as mentioned above, increases production of pro-myelopoietic cytokines by BM accessory cells. Moreover, with advancing age defective erythropoiesis is also observed that can progress to anemia [79, 80]. Here again, by releasing IL-1β and IL-18 from innate immunity cells, the Nlrp3 inflammasome may play a role, because, on the one hand, IL-1R signaling leads to a decrease in erythropoietin secretion in the kidney [81] and, on the other hand, IL-18 induces interferon gamma expression, which along with IL-1α synergistically inhibits erythroid colony formation [82].

The process responsible for these changes in hematopoiesis has recently been termed in the literature as “inflammaging”, which refers to chronic, low-grade, sterile inflammation that develops in hematopoietic tissues with advanced age [30]. This leads to the increased activity of innate immunity and a decrease in acquired immunity. These changes occur as a consequence of cellular turnover and chronic cellular stress in the absence of infection and are primarily driven by endogenous signals, including, besides SASP, cytokines; alarmines, such as eATP; uric acid crystals; oxidatively modified DNA; and aggregated proteins released from damaged cells [83]. All these DAMPs lead to activation of the Nlrp3 inflammasome, which most likely plays an important central role in BM aging. This enhanced basal level of inflammation may lead to an increased risk of clonal hematopoiesis of indeterminate potential, myeloid neoplasia, and spontaneous anemia, which will be discussed later in this review.

An important role in preventing aging in cells, including hematopoietic cells, is played by AMP-activated protein kinase (AMPK), which is a key energy sensor that regulates cellular metabolism to maintain energy homeostasis [84]. It is also involved in autophagy, which degrades and removes unnecessary or dysfunctional components of the cell, including damaged organelles, and allows their recycling. Autophagy also clears damaged mitochondria in a process referred to as mitophagy. Autophagic decline during aging results in the accumulation of cellular debris and damaged cellular organelles, whereas mitophagic decline during aging results in the accumulation of ROS and oxidized mitochondrial DNA, which has been shown to activate the Nlrp3 inflammasome [85, 86]. Thus, the decreases in autophagy and mitophagy occurring in aging hematopoietic cells lead to augmentation of the inflammaging response. Thus the autophagy process and the Nlrp3 inflammasome activation are inversely correlated, and inhibition of autophagy induces Nlrp3 inflammasome activation.

However, the basic question remains as to the exact role of the Nlrp3 inflammasome in inflammaging. Is it an initiator of this process, triggering activation of the innate immunity network? It would therefore be interesting to see, in well controlled experiments, whether Nlrp3-KO mice have an extended life span and/or improved hematopoietic parameters with age compared with normal control littermates. Similarly, the question of whether such mice would be resistant to myelodysplasia will be discussed later in this review. To our knowledge, such experiments have not been performed so far. On the other hand, as mentioned above, proper Nlrp3 inflammasome expression is required for maintaining the pool of normal HSPCs.

## Nlrp3 inflammasome as the culprit in “metaflammation” and involvement of the intracellular activation of complement “complosome”

The age-related inflammatory response discussed above can additionally be aggravated by an unhealthily modern lifestyle and excessive caloric consumption, which may lead to a pathophysiologic inflammatory response, as recent evidence indicates that the Nlrp3 inflammasome becomes activated by glucose or amino acid influx [87, 88]. On the one hand, this is not surprising, as there is a tight relationship between metabolism and the immune system, and all immunological processes depend on the energy supply; but on the other hand, excessive caloric uptake may lead to overactivation of the Nlrp3 inflammasome. The combined effect of inflammaging and related metabolic complications has been termed “metaflammation” [89–91].

Consistent with a role for the Nlrp3 inflammasome in cell metabolism [92], it has been shown that Nlrp3 is critical for the development of metabolic diseases, as Nlrp3 deficiency seems to have somewhat beneficial results in decreasing systemic inflammation, reducing immune cell activation, improving metabolism, and ameliorating resistance to insulin [87, 93–95]. On the other hand, a higher glucose level, as seen in type 2 diabetes patients, is associated with Nlrp3 inflammasome activation and increased serum levels of IL-1β and IL-18 [80, 96, 97].

The question then emerges of how to translate these observations directly to hematopoietic cells? In the context of metabolic regulation, an interesting interplay has emerged between the Nlrp3 inflammasome and intracellular activation of the ComC [47, 98]. It is well known that ComC proteins are synthesized and secreted into the circulation from the liver. However, recently it has been demonstrated somehow surprisingly that C3 and C5 proteins are also expressed in other cells [20–22]. Moreover, as demonstrated in T lymphocytes, C3 and C5 are cleaved to C3a and C5a anaphylatoxins, respectively, inside cells and interact with intracellular C3a and C5a receptors, C3aR and C5aR, respectively [20, 99, 100]. This intracellular signaling phenomenon has been described as the “complosome” and somewhat changes our view of the ComC, which was envisioned for many years as a serum-operative danger sensor and first-line-of-defense system [101]. However, this intracellular expression of C3 and C5 and their intracellular activation/cleavage to C3a and C5a fragments, which regulates the physiology of human T lymphocytes, sheds new light on the biological role of the ComC. As hypothesized recently, intracellular expression of C3 is an evolutionary remnant of its expression in single-cell organisms, where initially it was involved as a metabolism-regulating peptide [98]. It has been proposed that, during the evolution of multiorgan organisms, ComC proteins came to be synthesized in the liver, but some cells retained intracellular expression of C3. This concept is well discussed in a recent review [102]. An open question remains if the structure as well as the function of complement components synthesized in the liver and secreted into blood and their intracellular homologs might differ taking into consideration a possibility of posttranslational modification or enzymatic processing during synthesis inside cells [103].

Recently, activation of the ComC, activation of the C3aR and C5aR receptors by C3a and C5a, respectively, and activation of the complement regulator receptor CD46 by another C3 cleavage fragment, C3b opsonin, have been associated with the sensing of cell metabolic changes, such as increased amino acid influx and glycolysis involving mTORC1 [102, 103]. Based on the observation that an intracellular C3a fragment activates mTOR, an interesting concept has emerged explaining the presence in human T cells of the intracellular complement–metabolism–Nlrp3 inflammasome axis. Consistent with this concept, while intracellular C3a activates the cytoplasmic C3a receptor, C3b secreted from cells activates the CD46 receptor in an autocrine manner, and this coordinated action leads to activation of mTOR and increased glucose and amino acid uptake by membrane channels [102]. Thus, with respect to the phenomenon of metaflammation, mTORC1 has been identified as an Nlrp3 inflammasome activator [102].

The intracellular complosome activation signals C3a and C5a provide signal 1 for the transcription of Nrlp3 and IL-1β and signal 2 for increased oxygen metabolism and ROS production [101]. This process culminates in Nlrp3 inflammasome activation, IL-1β secretion, and optimal Th1 induction. Inhibition of mTOR by rapamycin abrogates Nlrp3 inflammasome activation and IL-1β production in T cells and reduces Th1 cell induction [102]. In parallel, intracellularly activated C5 and released C5a lead to an increase in ROS release from mitochondria, which additionally activates the Nlrp3 inflammasome and its biological effects [102].

**Fig. 5:**
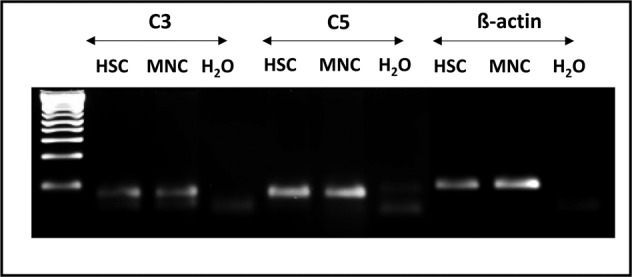
Expression of C3 and C5 mRNA in HSPCs. The expression of mRNA for the C3 and C5 genes was detected in purified mRNA in human CD34^+^ cells by reverse transcription polymerase chain reaction (RT-PCR). Samples containing only water instead of cDNA and samples without reverse transcriptase were used in each run as negative controls. Representative agarose gels of the RT-PCR amplicons are shown. Based on this, the potential role of the complosome in the biology of HSPCs requires further study.

This mechanism, which is primarily described for human T lymphocytes [102], is very likely to apply in other cells, including HSPCs. Consistent with this notion, we have recently detected expression of intracellular C3 and C5 in murine and human HSPCs (Fig. 5). Therefore, can the concept of a complosome and the role of the Nlrp3 inflammasome in metaflammation be extended to include the biology of HSPCs? To answer this question, however, will require extensive further investigation and such studies are undergoing in our laboratory.

## The Nlrp3 inflammasome in myelodysplastic syndrome (MDS), myeloproliferative neoplasms (MPN), and leukemia

### Myelodysplastic syndromes (MDS)

MDS are characterized by BM cytological dysplasia, a defect in the maturation of hematopoietic cells that results in ineffective hematopoiesis accompanied by recurrent somatic gene mutations and chromosomal abnormalities [104]. As currently understood, MDS is related to aberrant activation of innate immunity and a “smoldering” proinflammatory state in the hematopoietic microenvironment. An important function is at work here, as recent evidence indicates that activation of the Nlrp3 inflammasome potentiates BM inflammation and leads to hematopoietic cell damage, chromosomal abnormalities, expansion of BM myeloid-derived suppressor cells, and induction of pyroptosis in response to increased levels of extracellular DAMPs [105]. An important role as activators of the Nlrp3 inflammasome in the pathogenesis of MDS is played by the DAMP proteins S100A8 and S100A9, which are greatly increased in MDS patient blood plasma [106]. These proteins preferentially heterodimerize and deliver (i) NF-κB-mediated signal 1 after binding to TLR4 to enhance synthesis of Nlrp3 inflammasome complex proteins and (ii) activation of signal 2 after binding to CD33 antigen, which is expressed on hematopoietic cells.

Moreover, the S100A8/9 heterodimers induce pyroptosis in HSPCs in MDS patients [106]. In contrast to apoptosis, pyroptosis is lysis-based cell death and requires Nlrp3 inflammasome-mediated activation of caspase-1, which promotes insertion of the mature pore-forming protein gasdermin into the cell membrane [107]. This results in cell swelling, plasma membrane rupture, and massive release of IL-1β, IL-18, and intracellular DAMPs (eATP, HMGB1, DNA, and ASC oligomers). These factors together in the extracellular space recruit more immune cells and further perpetuate the inflammatory cascade in the BM microenvironment. Pyroptosis is in striking contrast to apoptosis, which is characterized by the packaging of cellular contents and non-inflammatory phagocytic uptake of membrane-bound apoptotic bodies [107]. Inhibition of S100A8/9-activated Nlrp3 inflammasome pathways are currently subject to pharmacological interventions by small-molecule inhibitors or antibodies. It was reported that either neutralization of S100A9 or inhibition of inflammasome machinery assembly prevented pyroptosis, restored proper clonogenicity of hematopoietic progenitors, and improved erythropoiesis in a S1009A transgenic mice model [106]. In future, more detailed investigations should be able to address whether, in addition to S1008/9 protein, other DAMPs, such as HMGB1, also contribute to the pathogenesis of MDS.

### Myeloproliferative neoplasms (MPS)

MPN include polycythemia vera, essential thrombocythemia, and myelofibrosis, and represent a unique model of the relationship between the clonal development of a hematologic malignancy and chronic inflammation. MPN are related to, and may evolve into, MDS or acute myeloid leukemia. It has been demonstrated that the MPN neoplastic clone drives this inflammatory reaction [108–110]. To support this, allogeneic stem cell transplantation leads not only to a complete restore of the hematopoiesis, regression of BM fibrosis, and a progressive healing of the chronic inflammation. Although chronic inflammation in BM microenvironment can be present before a malignant clone develops, most likely it represents a consequence of presence of malignant clone [109–111]. It is no doubt that Nlrp3 inflammasome plays an important role in pathogenesis and progression of MPN, however more detailed studies are needed to better address its role in this disorders, as it has been done already in case of MDS [106, 107].

### Leukemia

Chronic inflammation plays a supportive role in oncogenesis due to (i) promotion of genomic instability through DNA mutations and epigenetic changes, (ii) prevention of tumor immune surveillance, and (iii) predisposition to clonal evolution. Thus, the persistent chronic inflammation in BM and the presence of MDS or MPN may over time promote the development of leukemia. This process plays an important role in the pathogenesis of the leukemia seen in elderly patients as a consequence of inflammaging, which is driven, at least partially, by activation of the Nlrp3 inflammasome [30]. Another important aspect of leukemia and inflammation is the fact that inflammation in leukemic patients promotes release of several chemoattractants and thus increases trafficking of leukemic cells and their spread within hematopoietic organs. Our recent results indicate that the Nlrp3 inflammasome is an important driver of the migration and leukemic spread of leukemic cells [43].

## The Nlrp3 inflammasome in GvHD

Acute GvHD is a severe complication of hematopoietic transplantation, accompanied by high mortality rates, and the Nlrp3 inflammasome is clearly involved in its pathogenesis.

An important inducer of the Nlrp3 inflammasome in GvHD is activation of purinergic signaling by eATP [111–113]. After binding to the purinergic receptors P2X7 and P2X4, eATP induces activation of the Nlrp3 inflammasome [111]. However, this protein complex could also be activated by other DAMPs, cytokines, or active fragments of the ComC (C3a or C5a). An important DAMP that activates the Nlrp3 inflammasome after myeloablative conditioning for hematopoietic transplantation and that triggers GvHD is crystalized uric acid [114], the final breakdown product of purine metabolism released from ischemic tissues and dying cells [115].

It has been reported that IL-1β is released in an Nlrp3 inflammasome-dependent manner, which activates dendritic cells and T cells. In response to IL-1β, allogenic T cells differentiate into T helper 17 cells, which are a subset of the proinflammatory T helper cells implicated in autoimmune and inflammatory disorders, including the initiation of GvHD [116]. In agreement with experimental results in mice, enhanced levels of caspase-1 and IL-1β were also detected in circulating white blood cells and intestinal lesions in GvHD patients. These observations provide a new roadmap for adjuvant anti-GvHD therapy employing inhibitors of the Nlrp3 inflammasome, caspase-1, and caspase-1-activated IL-1β. In addition to IL-1β, another caspase-1 activation product, IL-18, has also been shown to increase in patients who develop acute GvHD after hematopoietic transplantation. The potential modulation of IL-18 in the pathogenesis of GvHD requires further study. However, an Nlrp3 inhibitor, MCC950, blocks, in addition to IL-1β release, IL-18 secretion, and the inflammatory type of cell death known as pyroptosis, as mentioned above.

## Conclusions

The Nlrp3 inflammasome has become an intriguing subject of studies on normal and pathological hematopoiesis. It is, as mentioned above, the best-studied member of the inflammasome family and shows several clear pleiotropic hematopoietic effects. On the other hand, several small-molecule modulators of Nlrp3 inflammasome signaling and appropriate KO mice are available. The number of publications related to the role of this interesting protein complex in maintaining homeostasis or its involvement in the pathogenesis of several disorders increases every month, and we can expect many new and interesting findings. One of the topics that needs to be addressed is the interplay between the Nlrp3 inflammasome and other members of this receptor family, which have both pro- and anti-inflammatory properties. [1, 2] More investigation is also needed to elucidate the mechanistic aspects of Nlrp3 inflammasome actions. Finally, in addition to the developed small-molecule inhibitor of the Nlrp3 inflammasome MCC950, potent new drugs are under investigation, and the first clinical trials in human are about to be launched.
